# Reproducibility of Novel Soft-Tissue Landmarks on Three-Dimensional Human Facial Scan Images in Caucasian and Asian

**DOI:** 10.1007/s00266-021-02642-4

**Published:** 2021-10-26

**Authors:** Zhouxiao Li, Riccardo Enzo Giunta, Konstantin Frank, Thilo Ludwig Schenck, Konstantin Christoph Koban

**Affiliations:** Division of Hand, Plastic and Aesthetic Surgery, University Hospital, LMU Munich, Pettenkoferstraße 8a, 80336 Munich, Germany

**Keywords:** Facial landmarks, Anthropometry, Three-dimensional surface imaging, Ethic and Gender

## Abstract

**Background:**

Three-dimensional surface imaging is established in many disciplines for objective facial acquisition regarding anthropometry. Former studies addressed the validation of landmark-based measurements for single race. In order to distinguish racial difference, the reproducibility of the landmark measurements must first be validated.

**Objectives:**

Our purpose is to validate the reproducibility of 46 facial soft-tissue landmarks on *x*, *y*, *z* axes to prove their reliability as 3D reference points.

**Methods:**

The study included 80 European Caucasian and 80 Chinese volunteers. Standardized 3D surface imaging was performed using Vectra 3D system. Two raters identified and defined 46 landmarks (138 coordinates), then repeatedly 3D-imaged volunteers' facial region in separate sessions. Coordinates' reproducibility of landmarks is divided into three categories (< 0.5 mm, < 1 mm, and >1 mm) for intra- and inter-rater reproducibility assessments.

**Results:**

Coordinates' reproducibility of 160 samples was distributed as follows: Intra-rater: < 0.5 mm (45%), < 1 mm (42%), >1 mm (13%); inter-rater: < 0.5 mm (31.2%), < 1 mm (42%), > 1 mm (26.8%). The reproducibility of landmarks in nasal tip region differs slightly between Caucasians and Asians. Compared to females, males typically have higher landmark reproducibility in lip and chin region. However, there were no differences in the reproducibility ranking of landmarks by gender.

**Conclusion:**

The majority of the 46 landmarks in the 3D plane are reproducible to 1 mm, which is clinically acceptable. All selected landmarks showed strong consistency across race and gender, suggesting their potential use as reference points in prospective clinical practice.

**Level of Evidence IV:**

This journal requires that authors assign a level of evidence to each article. For a full description of these Evidence-Based Medicine ratings, please refer to the Table of Contents or the online Instructions to Authors www.springer.com/00266.

**Supplementary Information:**

The online version contains supplementary material available at 10.1007/s00266-021-02642-4.

## Introduction

Three-dimensional surface imaging (3DSI) is an established means of facial analysis for infant facial development and congenital conditions such as cleft lip and palate or alterations of the skull, in facial reconstructive surgery, and for aesthetic facial plastic surgery consultation [1–3]. As a noninvasive technology, it also plays an increasingly important role in evaluating facial morphology. Surface imaging is often used in conjunction with computed tomography (CT) or magnetic resonance imaging (MRI).

Although there are numerous devices for three-dimensional (3D) acquisition, the basis of anthropometric surveying is often a landmark-based approach. Facial surface landmarks are critical to the accuracy of 3D facial morphology measurement and analysis [4, 5].

Currently, 3D cameras are promising tools for the assessment of facial soft-tissue morphology with high-resolution surface textures. The advantage lies in quickly collecting information and constructing high-resolution 3D images, which can accurately capture the subject's facial skin color and surface texture [6–8]. Rendered 3D models may provide benefit for (1) evaluation of pediatric facial development, (2) analysis of facial morphology of patients affected by congenital and acquired pathological factors, (3) facial analysis for patients undergoing facial cosmetic, reconstructive, and orthodontic procedures, and (4) morphological studies on the normalization of facial impairments [9–12].

Facial surface landmarks are critical to the accuracy of 3D morphological assessment. Although there has been much research on the reliability and reproducibility of landmarks on facial 3D images, they are mainly focused on one race and the sample sizes are relatively small. Additionally, many of them do not assess reproducibility, and most of the selected landmarks were traditional reference points that had been validated [7, 13–15]. Any facial morphological analysis based on anatomical landmarks requires highly reproductive and novel reference points across different patient populations. In this study, we aimed to define a number of novel facial soft-tissue landmarks to and assessed their reproducibility using a photogrammetric 3D stereophotography system

## Materials and Methods

### Study Sample

The study involved 80 European Caucasians (40 males, 40 females) and 80 Asian (40 males, 40 females). The age range of Caucasian volunteers was 20 to 50 years (30.49 ± 5.52 years). The age range of Asian volunteers was 18 to 45 years (30.36 ± 2.99 years). Written informed consent was obtained prior to enrollment. The study was approved by local university ethics committee (REF: 266-13) and conducted in accordance with the Declaration of Helsinki. Exclusion criteria were facial deformities, previous facial surgery, and volunteers diagnosed with epilepsy or other seizure disorders.

### 3D Stereophotography Equipment and Parameters

All facial 3d scans were captured by the high-resolution Vectra XT 3D Surface Imaging System (Canfield Inc., New Jersey, USA). It is a vertical fixed photography system with six integrated cameras at different angles. Its proprietary illumination system automatically adjusts the focus for optimal face imaging and its 3.5 mm photographic time reduces artifacts of the Vectra system due to the unconscious displacement of the subject.

### Image Sampling Process

Before taking the photograph, all volunteers were removed from any factors which would interfere with the image modeling: jewelry, glasses, and clothing elements (scarves, hat, etc.). They were asked to remove any hair from the face, forehead, and ears to completely expose the facial area. Male volunteers were asked to shave, while beards would cause image artifacts. The scan was performed in a well-lighted room. All volunteers were asked to sit in the same chair with a fixed backrest, lips kept closed without teeth grinding, and to look directly at the red marker dot on the 3D camera with a neutral facial expression and a natural head position. The lighting was kept under the same control conditions as in our daily work.

### 3D Processing

**Fig. 1:**
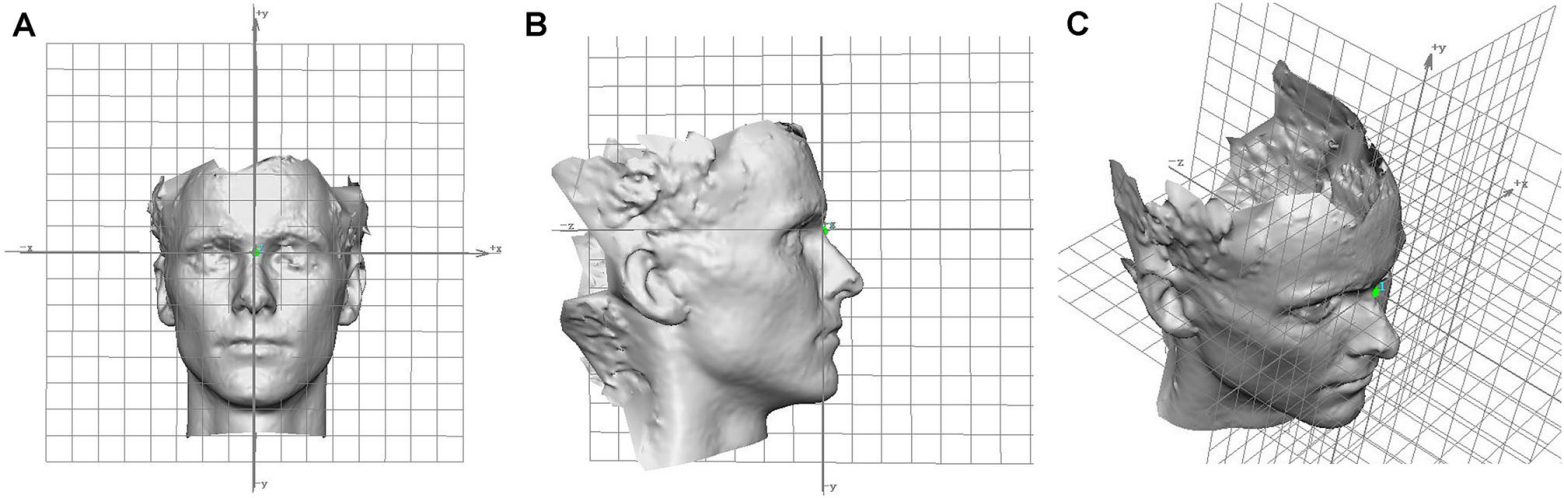
Standardized 3D facial images on three spatial planes

All captured images were processed, aligned, and analyzed using the proprietary Mirror® (Canfield Scientific; NJ, USA) [16]. The software was also implemented for providing reference frameworks with the *x*, *y*, *z* axis (sagittal, Y-Z plane; coronal, X-Y plane; and transverse, X-Z plane.) by unifying orientation for different images. The entire face was marked and symmetry of the planes was automatically adjusted by the integrated software. The midpoint between two endocanthions was chosen (mid-intercanthal point) as the origin point. The sagittal plane was referenced to the origin through the midline of the face, the coronal plane was determined to be the average natural head position, and the transverse plane was established to span the origin (Fig 1). The 3D face images were normalized on three planes to obtain comparable X, Y, and Z coordinates to assess the reproducibility of the facial landmarks [17].

### Data Evaluation

**Fig. 2:**
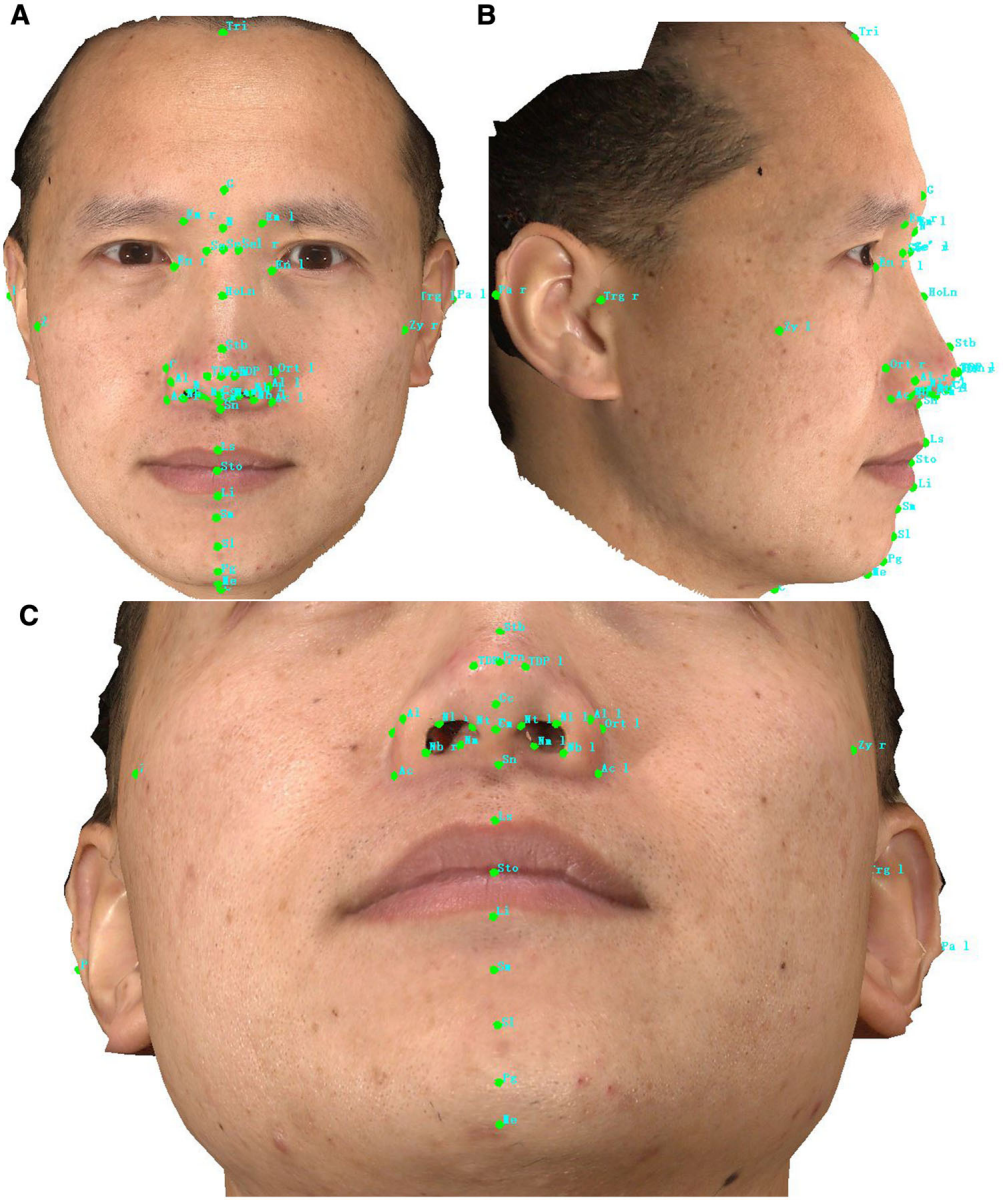
Nasal soft-tissue landmarks

In routine clinical facial surgery, surgeons will mark various anatomical landmarks for measurement. In this work, two raters identified and defined 46 landmarks based on former reports and the raters placed them separately on 3D facial images and all landmark coordinates were recorded (138 totally) for X,Y, Z axes [18–20]. Their name, abbreviation, and definition are displayed in Table 1. These landmarks contain some of the most commonly used classic landmarks and the novel landmarks that we found beneficial for facial measurements (Fig 2). Both raters are professional researchers in our department experienced in 3D anthropometry. One of the raters evaluates the reproducibility of all landmarks in a two-week interval to obtain intra-rater reproducibility. We then compared the two raters' results for inter-rater reproducibility. Based on the intra-rater and inter-rater assessment results of 160 volunteer, we calculated the mean and standard deviation for each landmark. The error of the landmarks is presented as the absolute difference in measurement of each landmark on three axes. It is divided into three categories (<0.5 mm, <1 mm, and >1 mm). Coordinates with a difference between the measurements of less than 0.5 mm of all samples are classified as highly reproducible; with a difference between 0.5 mm and 1 mm are determined to be moderately reproducible; with a difference above 1 mm are considered to be poorly reproducible [21, 22]. Both intra- and inter-rater evaluations assessed the reproducibility of landmarks of all samples on three planes, divided into race and gender.

**Table 1:** The name and definition of facial landmarks used in this study

Facial area	Landmarks	Definition
Eye	Em left	Lower margin of the left medial eyebrow end
	Em right	Lower margin of the right medial eyebrow end
	Endocanthion left (En l)	The left inner commissure of the palpebral fissure, the right midpoint of the frontonasal suture
	Endocanthion right (En r)	The right inner commissure of the palpebral fissure, the right midpoint of the frontonasal suture
Nose	Alar curvature/Alar crest left (Ac l)	Alar curvature point (ac) is the point located at the facial insertion of left alar base.
	Alar curvature/Alar crest right (Ac r)	Alar curvature point (ac) is the point located at the facial insertion of right alar base.
	Alare left (Al l)	The most lateral point on left alar contour
	Alare right (Al r)	The most lateral point on right alar contour
	Columella (Cm)	Most anterior and inferior point on apex of nose
	Columella constructed point (Cc)	the midpoint of the columella crest at the level of the nostril top points
	Glabella (G)	Most anterior point on midline of forehead
	Highnasal (Hn) or Lownasal (Ln)	The most anterior or posterior point on dorsum of nose between its root and tip
	Nasion (N)	Deepest point in middle of frontonasal curve
	Nostril base point left (Nb l)	The lowest point of each nostril or the inferior terminal point of left nostril axis.
	Nostril base point right (Nb r)	The lowest point of each nostril or the inferior terminal point of right nostril axis.
	Nostril lateral point left (Nl l)	The junction point of nostril short axis and the lateral margin of left nostril
	Nostril lateral point right (Nl r)	The junction point of nostril short axis and the lateral margin of right nostril
	Nostril medial point right (Nm l)	The junction point of nostril short axis and the medial margin of left nostril
	Nostril medial point right (Nm r)	The junction point of nostril short axis and the medial margin of right nostril
	Nostril top points left (Nt l)	The highest point of each nostril or the superior terminal point of left nostril axis.
	Nostril top points right (Nt r)	The highest point of each nostril or the superior terminal point of right nostril axis.
	Ort left	The left Junction of true vertical (TV) and true horizontal (TH) on the alare
	Ort right	the right Junction of true vertical (TV) and true horizontal (TH) on the alare
	Pronasale (Prn)	Most prominent point on apex of nose
	Sellion (Se)	The most posterior point of the frontonasal soft-tissue contour in the midline of the base of the nasal root.
	Sellion' left (Se' l)	The left intersections of TH[Se] and Dorsal aesthetic lines
	Sellion' right (Se' r)	The right intersections of TH[Se] and Dorsal aesthetic lines
	Subnasale (Sn)	Deepest point in nasolabial curvature
	Supratip break (Stb)	The joint point of the dorsum and nasal tip
	Tip defining point left(TDP l)	The left most anterior projection of the tip cartilages, usually corresponding to the apex of the lobular arch anatomically
	Tip defining point right (TDP r)	The right most anterior projection of the tip cartilages, usually corresponding to the apex of the lobular arch anatomically
Mouth	Cervical (C)	Deepest point at angel of chin and neck
	Labrale inferius (Li)	Lower lip vermilion border
	Labrale superius (Ls)	Upper lip vermilion border
	Menton (Me)	Most inferior point on inferior edge of chin
	Stomion(Sto)	The midpoint of the horizontal labial fissure
	Sublabiale(Sl)	The most posterior midpoint on the labiomental soft-tissue contour that defines the border between the lower lip and the chin.
	Supramental (Sm)	Deepest point in inferior sublabial concavity
	Pogonion (Pg)	Most anterior midpoint of chin
Ear	Postaurale left (Pa l)	Most posterior point on the free margin of the left ear
	Postaurale right (Pa r)	Most posterior point on the free margin of the right ear
	Tragus left (Trg l)	Most posterior point of auricular tragus left
	Tragus right (Trg r)	Most posterior point of auricular tragus right
Others	Trichion(Tri)	Intersection of hairline and midline of forehead
	Zygion left (Zy l)	The most lateral point on the outline of left zygomatic arch
	Zygion right (Zy r)	The most lateral point on the outline of right zygomatic arch

### Statistical Analysis

**Fig 3:**
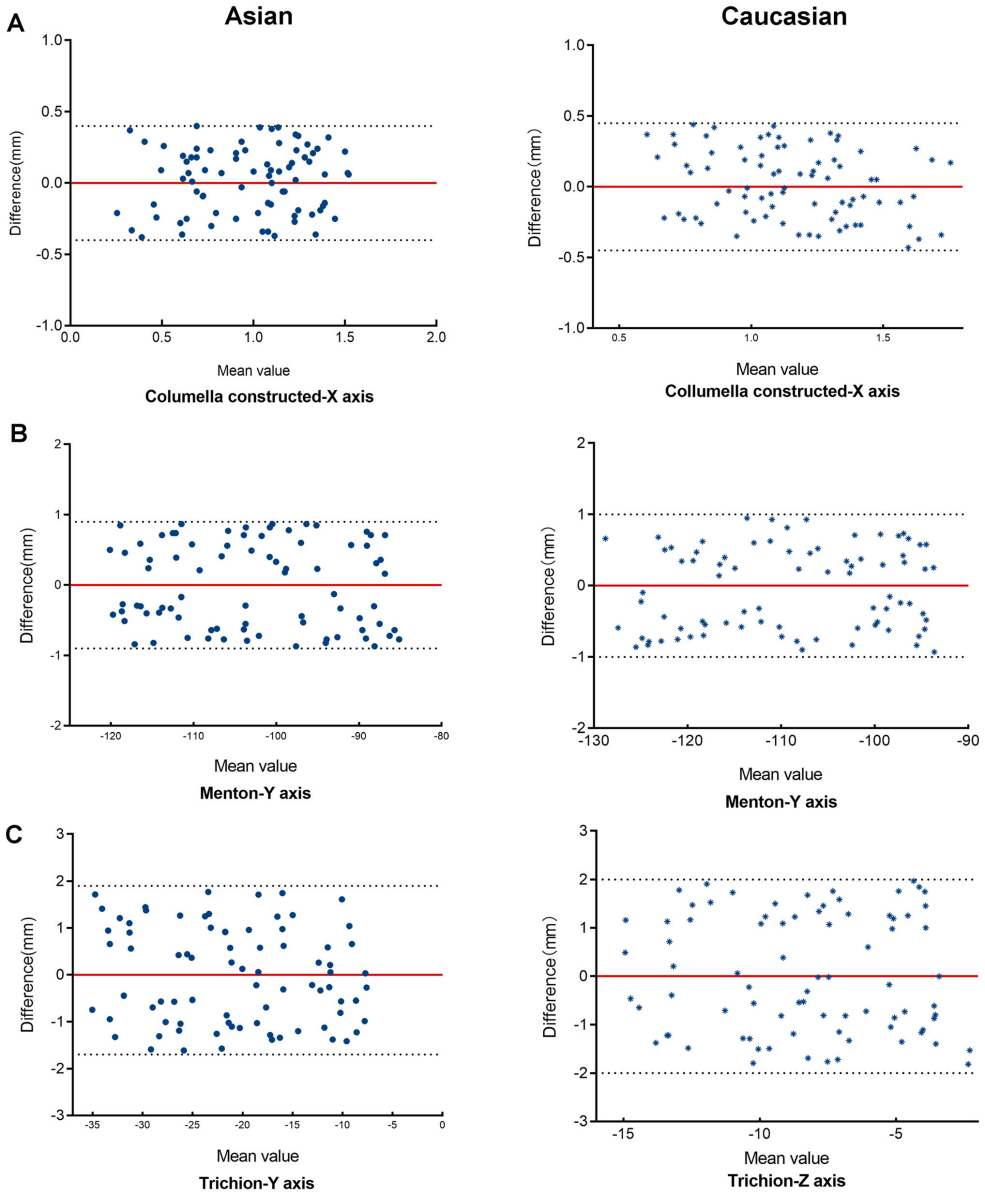
Reproducibility of representative landmarks identification between Caucasian and Asian (intra-rater)

**Fig. 4:**
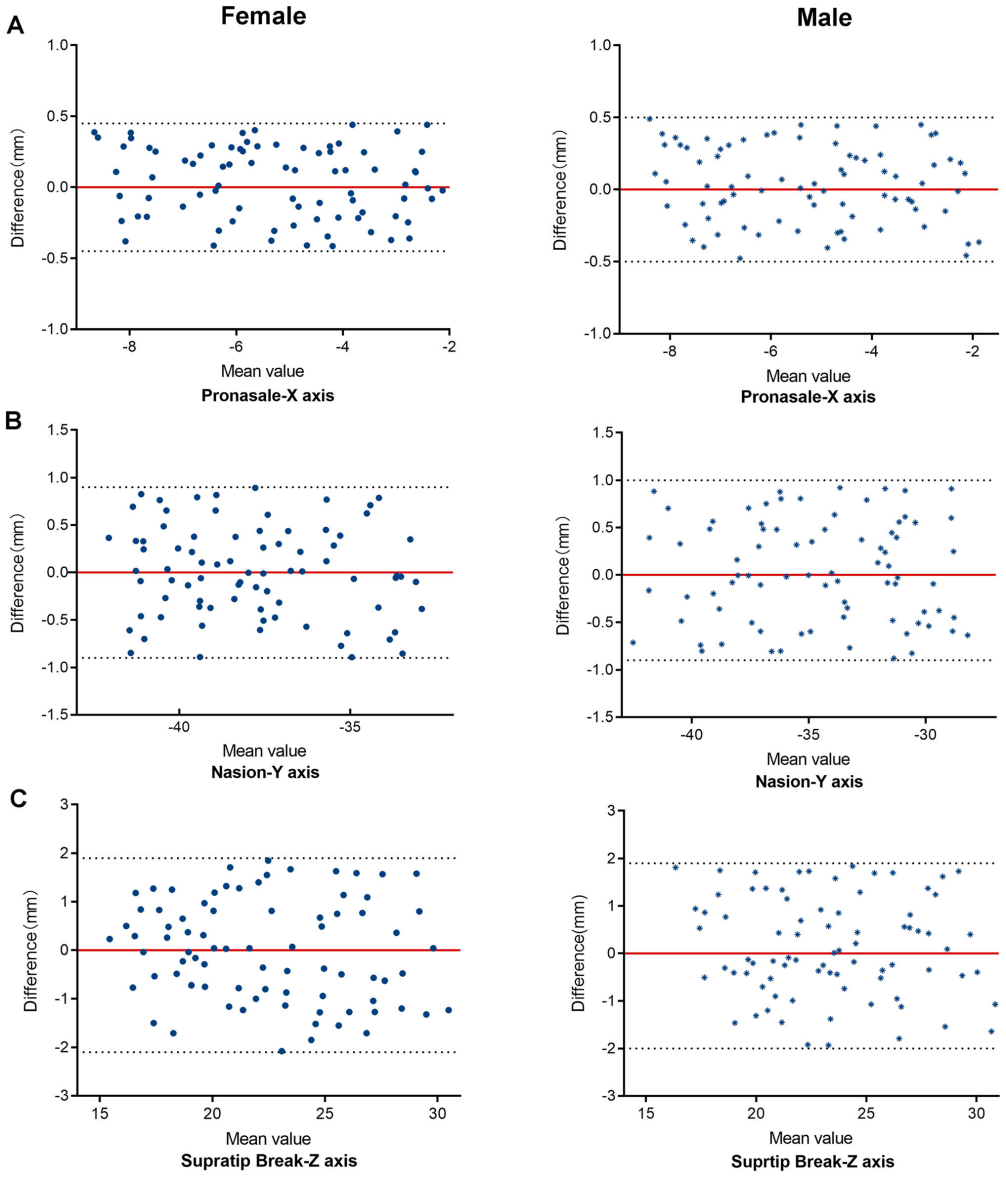
Reproducibility of representative landmarks identification between female and male (intra-rater)

A total of 88320 variables (46 landmarks×160 subjects×3 planes×2 raters×2 measurements) were analyzed with SPSS Statistics 23.00 (IBM, Armonk, NY, USA). Based on the absolute difference of each landmark on the x, y, and z axes, we calculate the total average reproducibility difference using the following formula to: $T=\sqrt{\frac{{(\Delta X)}^{2}+{(\Delta Y)}^{2}+{(\Delta Z)}^{2}}{3}}$, where *T* is the total average difference, $\Delta X$ is the difference on the x-axis, $\Delta Y$ is the difference on the y-axis, and $\Delta Z$ is the difference on the z axis. Each variable is first corrected to the median for all volunteers. Bland–Altman plots were carried out for intra- and inter-rater reproducibility assessments. For each plot, the difference between the measurements of each landmark coordinate was calculated and the average measurement for that particular coordinate is generated. In Figures 3 and 4, we exhibited the representative coordinates of selected landmarks to illustrate the consistency between high, moderate, and low levels of coordinates measurements in different scenarios. The vertical axis of Bland–Altman plots shows the measurement variance of the selected landmarks, while the horizontal axis shows the average of the measurements. The zero line refers to the subject with zero measurement variance (highest reproducibility). The two dashed lines above and below the zero line indicate the subject with the highest variance between the two measurement sessions.

## Results

### Overall Reproducibility

Table 2 shows the overall results of coordinates' reproducibility gained from intra- and inter-rater results of 160 volunteers. In addition to the overview, we display the results separately according to race and gender. Generally, reproducibility of most assessments was less than 1 mm (intra-examiner 87%, and inter-examiner 73.2%). In the Caucasian subgroups, the intra-examiner was 83.4% and inter-examiner was 71.1%; in Asian the intra-examiner was 79.7% and the inter-examiner was 72.5%. Among females, the intra-examiner was 79.7% and inter-examiner was 69.6%; among males, the intra-examiner was 82.6% and the inter-examiner was 73.2%. The highest reproducibility (<0.5 mm) coordinates were 45% (intra-rater) and 31.2% (inter-rater) of the 160 samples. The worst reproducibility (>1 mm) coordinates accounted for 13% (intra-rater) and 26.8% (inter-rater).

**Table 2:** Reproducibility of identified landmarks

Method of assessment	Intra-rater						Inter-rater					
Total	*n*=160						*n*=160					
Reproducibility level (mm)	<0.5		0.05≤d<1		d≥1		<0.5		0.05≤d<1		d≥1	
Number of coordinates	62		58		18		43		58		37	
Percentage (%)	45		42		13		31.2		42		26.8	

Race	Caucasian (*n*=80)			Asian (*n*=80)			Caucasian (*n*=80)			Asian (*n*=80)		
Reproducibility level (mm)	<0.5	0.05≤d<1	d≥1	<0.5	0.05≤d<1	d≥1	<0.5	0.05≤d<1	d≥1	<0.5	0.05≤d<1	d≥1
Number of coordinates	63	52	23	51	59	28	42	56	40	43	57	38
Percentage (%)	45.7	37.7	16.6	37	42.7	20.3	23.2	40.6	28.9	31.2	41.3	27.5

Gender	Female (*n*=80)			Male (*n*=80)			Female (*n*=80)			Male (*n*=80)		
Reproducibility level (mm)	<0.5	0.05≤d<1	d≥1	<0.5	0.05≤d<1	d≥1	<0.5	0.05≤d<1	d≥1	<0.5	0.05≤d<1	d≥1
Number of coordinates	58	52	28	62	52	24	41	55	42	42	59	37
Percentage (%)	42	37.7	20.3	44.9	37.7	17.4	29.7	39.9	30.4	30.4	42.8	26.8

Total number of coordinates = 138

The error results of all landmarks on the x, y, and z axes after all samples were grouped by gender and race are shown in Tables 3 and 4, respectively (mean and SD). Additionally, we calculated the total error based on the results on each axis for each landmark. The landmarks were ranked from most reproducible to least reproducible for both intra- and inter-examiner assessments. Compared to the intra-rater assessments, we noticed that the corresponding inter-rater results showed poorer reproducibility.

**Table 3:** Ranking of facial soft-tissue landmarks in Caucasian and Asian in respect to their reproducibility in the three spatial planes

Race	Caucasian (*n*=80)					Asian (*n*=80)				
Area	Landmarks	Intra-rater		Inter-rater		Landmarks	Intra-rater		Inter-rater	
		Mean	SD	Mean	SD		Mean	SD	Mean	SD
Nose	Nl l	0.17	0.14	0.34	0.23	Nl l	0.20	0.15	0.25	0.22
	Nl r	0.17	0.12	0.43	0.45	Nl r	0.24	0.21	0.28	0.26
	Nb l	0.20	0.14	0.48	0.49	Nb r	0.25	0.17	0.30	0.23
	Nb r	0.21	0.26	0.48	0.15	Nb l	0.27	0.30	0.38	0.28
	Nt r	0.22	0.16	0.51	0.46	Nt r	0.30	0.27	0.39	0.36
	Nt l	0.23	0.15	0.54	0.27	Nt l	0.43	0.36	0.49	0.34
	Nm l	0.25	0.19	0.55	0.33	Cc	0.43	0.38	0.54	0.42
	Nm r	0.26	0.19	0.55	0.49	Cm	0.43	0.28	0.60	0.47
	G	0.31	0.21	0.61	0.38	G	0.44	0.38	0.63	0.48
	Prn	0.31	0.21	0.71	0.55	Prn	0.44	0.32	0.63	0.52
	Cm	0.35	0.24	0.72	0.53	Al l	0.45	0.57	0.65	0.53
	Stb	0.39	0.29	0.79	0.56	Al r	0.58	0.46	0.71	0.44
	TDP r	0.44	0.40	0.84	0.65	TDP r	0.59	0.51	0.71	0.57
	TDP l	0.44	0.39	0.85	0.62	TDP l	0.65	0.55	0.74	0.69
	Se	0.48	0.40	0.88	0.75	Se	0.68	0.63	0.76	0.58
	Cc	0.54	0.39	0.92	0.55	Nm r	0.69	0.50	0.77	0.63
	Ac l	0.64	0.47	0.93	0.72	Nm l	0.71	0.60	0.78	0.57
	Holn	0.67	0.47	1.00	0.69	Holn	0.78	0.70	0.98	0.73
	Ac r	0.68	0.50	1.01	0.75	Ac r	0.86	0.67	0.99	0.71
	Se'r	0.69	0.54	1.03	0.72	Se'r	0.93	0.51	1.00	0.82
	Se'l	0.72	0.69	1.06	0.74	Se'l	0.94	0.75	1.07	0.94
	N	0.73	0.44	1.07	0.50	N	0.95	0.60	1.08	0.86
	Al l	0.75	0.61	1.09	0.68	Ac l	0.95	0.46	1.09	0.51
	Al r	0.75	0.55	1.11	0.64	Stb	1.04	0.62	1.10	0.71
	Sn	0.83	0.57	1.15	0.58	Sn	1.04	0.86	1.11	0.83
	Ort l	0.90	0.60	1.18	0.73	Ort l	1.04	0.97	1.13	0.78
	Ort r	0.94	0.66	1.20	0.87	Ort r	1.11	0.69	1.13	0.74
Eye	En r	0.44	0.31	0.49	0.35	En r	0.30	0.22	0.54	0.41
	En l	0.49	0.34	0.65	0.51	En l	0.38	0.30	0.67	0.53
	Em r	0.54	0.30	0.66	0.30	Em r	0.61	0.46	0.77	0.47
	Em l	0.61	0.55	0.95	0.72	Em l	0.62	0.57	0.85	0.55
Mouth	Sto	0.43	0.27	0.52	0.43	Sto	0.47	0.27	0.64	0.57
	Li	0.43	0.36	0.58	0.32	Li	0.49	0.51	0.67	0.43
	Ls	0.44	0.33	0.60	0.36	Ls	0.61	0.47	0.68	0.52
	Sm	0.60	0.45	0.66	0.61	Sm	0.64	0.47	0.81	0.52
	Sl	0.75	0.46	0.85	0.50	Sl	0.86	0.64	0.95	0.48
	Pg	0.98	0.72	1.45	0.65	Pg	1.21	1.03	1.31	0.78
	Me	1.11	0.91	1.54	1.25	Me	1.50	1.14	1.63	1.03
	C	1.47	0.84	1.75	1.22	C	1.52	0.94	1.73	0.69
Ear	Trg l	0.72	0.54	1.19	1.19	Trg l	0.86	0.73	1.05	0.74
	Trg r	0.87	0.68	1.29	0.67	Trg r	0.88	0.76	1.22	0.88
	Pa l	0.90	0.63	1.59	0.65	Pa l	0.89	0.55	1.29	0.92
	Pa r	1.07	0.69	1.63	0.68	Pa r	0.97	0.89	1.40	0.90
Others	Tri	1.52	1.10	1.94	1.05	Tri	1.23	0.95	1.43	0.99
	Zy r	1.62	0.97	2.00	1.02	Zy r	2.01	1.08	2.12	1.30
	Zy l	1.79	1.38	2.59	1.19	Zy l	2.04	1.25	2.21	1.12

**Table 4:** Ranking of facial soft-tissue landmarks in females and males in respect to their reproducibility in the three spatial planes

Gender	Female(*n*=80)					Male (*n*=80)				
Area	Landmarks	Intra-rater		Inter-rater		Landmarks	Intra-rater		Inter-rater	
		Mean	SD	Mean	SD		Mean	SD	Mean	SD
Nose	Prn	0.24	0.22	0.32	0.21	Prn	0.21	0.19	0.31	0.19
	Cm	0.25	0.21	0.34	0.27	Cm	0.23	0.20	0.32	0.14
	Holn	0.25	0.16	0.35	0.26	Holn	0.23	0.17	0.33	0.14
	TDP r	0.30	0.22	0.35	0.25	TDP r	0.27	0.19	0.36	0.20
	TDP l	0.34	0.91	0.56	0.48	TDP l	0.30	0.53	0.52	0.43
	Se	0.38	0.38	0.59	0.19	Se	0.31	0.32	0.52	0.36
	G	0.39	0.30	0.70	0.55	G	0.32	0.24	0.57	0.45
	Ac l	0.38	0.93	0.77	0.52	Ac l	0.36	0.45	0.59	0.53
	Ac r	0.42	0.23	0.77	0.52	Ac r	0.38	0.32	0.72	0.62
	Nt l	0.43	0.33	0.79	0.57	Nt l	0.39	0.22	0.72	0.47
	Nt r	0.46	0.40	0.83	0.55	Nt r	0.40	0.23	0.75	0.48
	Nl l	0.47	0.43	0.84	0.59	Nl l	0.46	0.46	0.89	0.64
	Nl r	0.54	0.47	0.93	0.75	Nl r	0.49	0.53	0.95	0.84
	Ort l	0.57	0.44	0.94	0.62	Ort l	0.52	0.30	1.03	0.94
	Ort r	0.59	0.30	0.95	0.61	Ort r	0.53	0.36	1.04	0.74
	Cc	0.65	0.41	0.95	0.75	Cc	0.62	0.54	1.12	1.03
	Sn	0.66	0.58	0.85	0.56	Sn	0.64	0.57	0.98	0.56
	Se'l	0.66	0.55	1.04	0.64	Se‘l	0.65	0.66	1.16	0.91
	Se'r	0.71	0.57	1.08	0.65	Nb l	0.73	0.49	1.20	0.49
	Nb l	0.73	0.80	1.13	0.63	Nb r	0.73	0.61	1.25	0.86
	Nb r	0.75	0.65	1.21	1.01	Se'r	0.75	0.51	1.34	0.99
	Nm l	0.80	0.56	1.30	0.48	Nm l	0.79	0.57	1.35	1.00
	Nm r	0.84	0.54	1.30	0.83	Nm r	0.80	0.73	1.42	0.75
	Al r	0.85	0.56	1.57	1.02	Al r	0.85	0.62	1.50	1.12
	Al l	0.86	0.80	1.61	1.03	Al l	0.86	0.54	1.51	1.01
	N	0.90	0.60	1.52	0.69	N	0.99	0.84	1.35	0.89
	Stb	1.55	1.01	1.88	0.91	Stb	1.45	0.92	1.65	0.76
Eye	Em l	0.31	0.26	0.45	0.35	Em l	0.38	0.29	0.49	0.36
	Em r	0.39	0.36	0.54	0.35	Em r	0.43	0.32	0.56	0.53
	En r	0.48	0.40	0.71	0.60	En r	0.67	0.50	0.79	0.82
	En l	0.53	0.44	0.76	0.67	En l	0.68	0.55	0.85	0.48
Mouth	Ls	0.43	0.36	0.53	0.42	Ls	0.41	0.29	0.50	0.70
	Li	0.52	0.48	0.62	0.32	Li	0.44	0.28	0.52	0.45
	Sto	0.59	0.46	0.68	0.50	Sto	0.45	0.40	0.54	0.27
	Me	0.60	0.55	0.82	0.59	Me	0.50	0.45	0.68	0.49
	Sl	0.72	0.60	1.04	0.81	Sl	0.70	0.54	0.99	0.58
	Pg	0.73	0.41	1.09	1.19	Pg	0.70	0.36	1.04	0.75
	Sm	1.13	1.01	1.51	1.04	Sm	1.02	0.71	1.36	0.73
	C	1.33	0.83	1.83	1.17	C	1.08	0.82	1.57	0.99
Ear	Pa l	0.79	0.52	1.40	1.07	Pa l	0.78	0.64	1.12	0.66
	Pa r	0.87	0.71	1.65	0.95	Pa r	0.84	0.65	1.13	0.66
	Trg r	1.04	0.63	1.42	0.62	Trg r	0.87	0.66	1.15	0.68
	Trg l	1.22	0.68	1.99	0.93	Trg l	1.14	0.97	1.45	0.91
Others	Tri	1.53	1.07	1.64	0.95	Tri	1.50	0.93	1.58	0.89
	Zy r	1.87	1.35	2.09	1.27	Zy r	1.64	1.06	1.92	0.99
	Zy l	1.97	1.49	2.25	1.36	Zy l	1.77	1.17	2.27	0.82

### The Reproducibility of Landmarks in Caucasian and Asian Participants

Landmark accuracy for the Caucasian group ranged from 0.17 to 0.94 mm (intra-rater) and 0.20 to 1.38 mm (inter-rater) in the nose area, 0.44 to 0.61 mm (intra-rater) and 0.49 to 0.95 mm (inter-rater) in the eye area, 0.44 to 1.47 mm (intra-rater) and 0.52 to 1.75 mm (inter-rater) in the mouth area, 0.72 to 1.07 mm (intra-rater) and 1.19 to 1.63 mm (inter-rater) in the ear area, and 1.52 to 1.79 mm (intra-rater) and 1.23 to 2.04 mm (inter-rater) in the other areas.

Landmark accuracy for the Asian group ranged from 0.20 to 1.38 mm (intra-rater) and 0.25 to 1.13mm (inter-rater) in the nose area, 0.30 to 0.62 mm (intra-rater) and 0.54 to 0.85 mm (inter-rater) in the eye area, 0.47 to 1.52 mm (intra-rater) and 0.67 to 1.73 mm (inter-rater) in the mouth area, 0.86 to 0.97 mm (intra-rater) and 1.05 to 1.40 mm (inter-rater) in the ear area, and 1.23 to 2.04 mm (intra-rater) and 1.43 to 2.21 mm (inter-rater) in the other areas.

Differences in landmark reproducibility between Caucasian and Asian were concentrated in nose tip, alare, and nostril area, including Sn, Cc, Cm, Al right, Al left, Ac right, Nm of both sides, and Stb point. Several landmarks showed poor reproducibility in both Caucasians and Asians, namely Tri, Zy right, and Zy left. The landmarks with the most significant intra- and inter-group differences were Zy left with 0.8 mm in Caucasians and Pa right with 0.43 mm in Asians. Moreover, the measurement differences between intra- and inter-group assessment of landmarks in the Asian group were generally smaller than in the Caucasian group. The most and least reproducible landmarks in Asians were consistent with those in Caucasians **(**Table 3 & Supplement Table 1).

### The Reproducibility of Landmarks in Female and Male Subgroups

The accuracy of landmarks in the female subgroup ranged from 0.24 to 1.55 mm (intra-rater) and 0.32 to 1.88 mm (inter-rater) in the nose area, 0.31 to 0.53 mm (intra-rater) and 0.45 to 0.76 mm (inter-rater) in the eye area, 0.40 to 1.33 mm (intra-rater) and 0.53 to 1.83 mm (inter-rater) in the mouth area, 0.79 to 1.22 mm (intra-rater) and 1.40 to 1.99 mm (inter-rater) in the ear area, and 1.19 to 1.97 mm (intra-rater) and 1.17 to 2.25 mm (inter-rater) in the other areas.

The accuracy of landmarks in the male subgroup ranged from 0.21 to 1.45 mm (intra-rater) and 0.31 to 1.65 mm (inter-rater) in the nose area, 0.38 to 0.68 mm (intra-rater) and 0.49 to 0.85mm (inter-rater) in the eye area, 0.41 to 1.08 mm (intra-rater) and 0.50 to 1.57 mm (inter-rater) in the mouth area, 0.83 to 1.14 mm (intra-rater) and 1.20 to 2.06 mm (inter-rater) in the ear area, and 1.28 to 1.45 mm (intra-rater) and 1.03 to 2.43 mm (inter-rater) in the other areas.

Compared to females, landmarks concentrating on the nose and mouth areas had higher reproducibility in males in intra-rater, while landmarks in the eye area had poorer reproducibility in males. Moreover, the deviations between intra- and inter-rater in males were smaller than in females.

We did not notice significant differences in the ranking of landmark reproducibility between genders overall, except for the Sellion right and Nostril base point left and right. Among both female and male groups, Pronasale (prn) was the most reproducible landmark, while Zygion left was the least reproducible landmark (Table 4 and Supplement Table 1).

Some landmarks differed in the reproducibility levels in intra-rater and inter-rater assessments as follows: Cm and Stb were highly reproducible (<0.5 mm) in intra-rater and moderately (<1 mm) in inter-rater assessment for the Caucasian sample. In Asian sample, Se right and Se left were moderately reproducible (<0.5 mm) in intra-rater and poorly reproducible (>1 mm) in inter-rater assessment.

### The Representative Landmarks in Ethnic and Gender Subgroups

Bland–Altman plots are used to illustrate the consistency level between the values of each 3D coordinate (X, Y, and Z) for the facial landmarks. Some representative coordinates of facial landmarks are given in Fig. 3 to illustrate the high, moderate, and low levels of consistency between the measurements obtained from intra-rater assessment of the ethnic subgroup. Figure 3a indicates that the landmark Columella (Cm) was highly reproducible (<0.5 mm) in the X-plane for Caucasians and Asians. Figure 3b indicates that the landmark Menton (M) was moderately reproducible (>0.5 mm) in the Y-plane. Figure 3c indicates that the landmark Trichion (Tri) was poorly reproducible (>1 mm) in the Z-plane. Figure 4 exhibits some representative coordinates of the measurements obtained from the inter-rater assessment for facial landmarks in gender subgroup. Figure 4a indicates that the landmark Pronasale (Prn) was highly reproducible (<0.5 mm) in the X-plane for both females and males. Figure 4b indicates that the landmark Nasion (N) was moderately reproducible (>0.5 mm) in the Y-plane. Figure 4c indicates that the landmark Supratip break point (Stb) was poorly reproducible in the Z-plane (>1 mm).

## Discussion

Facial soft-tissue landmarks and their anthropometric measurements play an important role in the clinical practice of numerous medical disciplines, particularly in reconstructive and aesthetic plastic surgery, otorhinolaryngology along with oral and maxillofacial surgery. Landmark-based cephalometric measurements facilitate diagnosis, counseling, and treatment planning, as well as an objective evaluation of a therapeutic outcome. The reproducibility of facial soft-tissue landmarks has been studied in detail on 2D photography and several classic facial landmarks have been validated for their utility in 3D surface imaging [15, 21, 23].

In the current study, we identified several non-traditional facial soft landmarks based on daily clinical experience and validated all 46 landmarks in gender-identical Caucasian and Asian samples. We explored the reproducibility of these soft-tissue landmarks on 3D facial images of our two ethnic groups. The reproducibility of facial landmarks has been validated in the three spatial planes and our measurements showed that the majority coordinates in *x*, *y*, *z* axes of the 46 landmarks are reproducible to less than 1 mm, which is clinically acceptable (87% intra-rater and 73.2% inter-rater). The reproducibility of the intra-rater evaluation was higher than that of the inter-rater.

Based on our measurements in different intra-rater and inter-rater sessions, we inferred the following criteria involved in facial landmarks' reproducibility. First, the clear description and definition of landmarks. Second, the morphology and contour of the facial area in which the landmark is located. Landmarks located in more projecting or well-defined areas have a higher reproducibility. For instance, Prn and TDP are more reproducible than Zy, which is located on a flatter site. Third, features and characteristics of landmarks. Landmarks with distinctive features usually have a higher reproducibility. Four, the ethnicities and genders with different facial features and characteristics. Five, rater dependency. Examples include level of attention, discipline and consistency, proficiency in 3D imaging software and knowledge of facial anatomy. Six, the quality of 3D imaging. The landmarks on the artifact-free and defect-free areas are more reproducible.

In addition, the reproducibility of our landmarks varies in the three planes. For example, certain landmarks are harder to locate accurately on one axis than on the other two. Previous studies have reported similar results [24, 25]. Medelnik et al. attribute this bias to the position of landmark relative to the individual's facial morphology [26]. The poorly reproducible landmarks are mostly concentrated on nose alare, chin, Trichion, and Zygion. Raters may not be able to find a suitable reference point in less clearly demarcated areas. Hair-bearing skin areas, such as the hairline, usually have lower reproducibility [27]. Moreover, it has been reported that the patient's head occasionally needs to be tilted back slightly to ensure data quality in nose and chin area, which complicates ensuring a consistent recording position [28]. Therefore, precautions should be taken in the preparation of the 3DSI to minimize hair and sitting-induced errors and to make landmark identification more precise.

Some landmarks and coordinates vary by race. Landmarks distributed in the nasal tip and nostrils, such as Nl, Nb, Nt, Nm, TDP, Stb, and Prn, are more reproducible in Caucasians. Nasal anatomical features of Caucasians differ from those of Asians. A previous study found that the Caucasian descent typically has relatively thick nasal skin, straighter dorsum, more pronounced nasal tip, and teardrop shape nostrils. Correspondingly, the bony vault in Asians is usually wide and short. The dorsal aesthetic lines were not clearly defined, resulting in a less well-defined TDP. The nasal tip was widened with wide alar bases. The nasal length was shortened, with diminished tip projection and horizontally oriented nostrils [29]. These factors could impact the raters to identify the nasal tip and the nostril axis and to locate the landmarks associated with these regions in Caucasians. Thus, before clinical use of a 3D landmark-based study, the reliability of the measurements for different ethnicities would need to be investigated separately.

We also observed slight gender-dependent differences in landmark placement accuracy. In intra-and inter-rater reproducibility assessment, the landmark Zy on both sides of the x-axis produced fewer errors in males. Our observation that males have larger and more pronounced zygomatic bone than females, which facilitates the rater to position Zy on the x-axis. Previous anthropometric studies have shown that males have more angular chin and jawbones than females. Male's jaws are on average 17% higher vertical and have more lateral fullness, which may affect the 3D placement of landmarks in these areas [30]. These features may make it easier to locate the landmarks Ls, Li, Me, and C.

In terms of device technology, it should be noted that Vectra XT 3D Surface Imaging System has some imperfections in 3D modeling of hair-bearing skin areas and complex structures, making it prone to artifacts and distortions. Nevertheless, despite its limitations, photogrammetry still has an irreplaceable role and potential for widespread application in predicting soft-tissue contours and monitoring treatment progress, especially for patients undergoing complex rhinoplasty and maxillofacial plastic surgery, as well as consultations for orthodontic treatment or orthognathic surgery.

## Conclusion

Before being widely used in clinics, the reproducibility of each facial landmark should be verified on the x, y, and z three planes. In order to obtain good reproducibility, the rater placing landmarks must clearly define and thoroughly understand their definitions. Landmarks located at different positions on the face have broad variation in reproducible levels; the landmarks placed on clear features and boundaries area have higher reproducibility than those placed on flat or a gently curved plane. This may be related to gender and ethnic differences in facial morphology, leading to variations in the reproducibility of certain landmarks. It is also essential for raters to have sufficient knowledge of facial anatomy and proficiency in 3D images to improve the reproducibility of landmarks. In this study, the majority of the 138 coordinates from 46 facial landmarks had a reproducibility of less than 1 mm, which is clinically acceptable (87% intra-examiner and 73.2% inter-examiner). Therefore, 3D scanning with Vectra XT 3D Surface Imaging System meets the requirement of cephalometry based on facial soft-tissue landmarks in daily clinical practice. Meanwhile, race and gender reproducibility bias of the different landmarks should be taken into account during the evaluation.

## Supplementary Information

Below is the link to the electronic supplementary material.

Supplementary file1 (DOCX 87 KB)

## Declarations

### Informed consents

Written informed consents were obtained from all participants correspond with the Declaration of Helsinki protocols (1996). This study was performed in line with local laws and good clinical practice (1996) and approved by the Ethics Committee of the Ludwig-Maximilians-University Munich (Reference Number 266-13).
