# Supplementary material for: Reproducibility of Novel Soft-Tissue Landmarks on Three-Dimensional Human Facial Scan Images in Caucasian and Asian
Source: Aesthetic Plast Surg. 2021 Oct 26;46(2):719–31. doi: 10.1007/s00266-021-02642-4 (PMC9090709; doi:10.1007/s00266-021-02642-4)
Supplement: Supplementary file 1 — Supplementary file1 (DOCX 87 KB) [file 266_2021_2642_MOESM1_ESM.docx]

**Supplementary Table 1. Overall evaluation of the reproducibility of Asian and Caucasian facial soft tissue landmarks on three spatial planes**

| Race | Caucasian (n=80) | | | | | | Asian (n=80) | | | | | | Race | Caucasian (n=80) | | | | | | Asian (n=80) | | | | | |
| --- | --- | --- | --- | --- | --- | --- | --- | --- | --- | --- | --- | --- | --- | --- | --- | --- | --- | --- | --- | --- | --- | --- | --- | --- | --- |
| Area | Landmarks | Axis | Intra-rater | | Inter-rater | | Landmarks | Axis | Intra-rater | | Inter-rater | | Area | Landmarks | Axis | Intra-rater | | Inter-rater | | Landmarks | Axis | Intra-rater | | Inter-rater | |
|  |  |  | Mean | SD | Mean | SD |  |  | Mean | SD | Mean | SD |  |  |  | Mean | SD | Mean | SD |  |  | Mean | SD | Mean | SD |
| Nose | Nl l | x | 0.15 | 0.15 | 0.42 | 0.26 | Nl l | x | 0.30 | 0.21 | 0.30 | 0.25 | Nose | Al r | x | 0.68 | 0.54 | 0.98 | 0.43 | Stb | x | 0.56 | 0.52 | 0.84 | 0.60 |
|  |  | y | 0.13 | 0.12 | 0.22 | 0.14 |  | y | 0.09 | 0.08 | 0.21 | 0.20 |  |  | y | 0.76 | 0.51 | 1.15 | 0.64 |  | y | 1.67 | 0.86 | 1.29 | 0.84 |
|  |  | z | 0.22 | 0.15 | 0.35 | 0.28 |  | z | 0.16 | 0.12 | 0.22 | 0.21 |  |  | z | 0.80 | 0.60 | 1.19 | 0.78 |  | z | 0.39 | 0.37 | 1.13 | 0.66 |
|  | Nl r | x | 0.19 | 0.13 | 0.32 | 0.58 | Nl r | x | 0.22 | 0.19 | 0.32 | 0.29 |  | Sn | x | 0.53 | 0.31 | 0.86 | 0.38 | Sn | x | 1.03 | 0.66 | 0.63 | 0.47 |
|  |  | y | 0.16 | 0.10 | 0.43 | 0.36 |  | y | 0.28 | 0.24 | 0.31 | 0.31 |  |  | y | 1.16 | 0.86 | 1.46 | 0.57 |  | y | 1.47 | 1.31 | 1.46 | 1.12 |
|  |  | z | 0.15 | 0.12 | 0.52 | 0.39 |  | z | 0.21 | 0.18 | 0.18 | 0.13 |  |  | z | 0.67 | 0.37 | 1.04 | 0.74 |  | z | 0.19 | 0.28 | 1.09 | 0.77 |
|  | Nb l | x | 0.24 | 0.16 | 0.50 | 0.46 | Nb r | x | 0.28 | 0.17 | 0.31 | 0.30 |  | Ort l | x | 0.71 | 0.65 | 0.84 | 0.58 | Ort l | x | 1.13 | 0.89 | 0.55 | 0.66 |
|  |  | y | 0.20 | 0.14 | 0.41 | 0.61 |  | y | 0.20 | 0.13 | 0.20 | 0.19 |  |  | y | 1.22 | 0.65 | 1.65 | 0.93 |  | y | 1.25 | 1.37 | 1.71 | 0.99 |
|  |  | z | 0.13 | 0.10 | 0.53 | 0.39 |  | z | 0.25 | 0.21 | 0.37 | 0.19 |  |  | z | 0.66 | 0.49 | 0.88 | 0.63 |  | z | 0.66 | 0.42 | 0.78 | 0.64 |
|  | Nb r | x | 0.19 | 0.16 | 0.55 | 0.15 | Nb l | x | 0.32 | 0.35 | 0.38 | 0.22 |  | Ort r | x | 0.61 | 0.48 | 1.04 | 0.91 | Ort r | x | 0.76 | 0.59 | 1.27 | 0.69 |
|  |  | y | 0.26 | 0.26 | 0.45 | 0.17 |  | y | 0.24 | 0.32 | 0.30 | 0.25 |  |  | y | 1.32 | 0.89 | 1.52 | 1.08 |  | y | 1.39 | 0.84 | 1.20 | 0.86 |
|  |  | z | 0.17 | 0.34 | 0.42 | 0.13 |  | z | 0.26 | 0.21 | 0.45 | 0.35 |  |  | z | 0.72 | 0.53 | 0.97 | 0.52 |  | z | 1.08 | 0.61 | 0.88 | 0.65 |
|  | Nt r | x | 0.25 | 0.16 | 0.39 | 0.21 | Nt r | x | 0.37 | 0.37 | 0.26 | 0.18 | Eye | En r | x | 0.43 | 0.27 | 0.45 | 0.32 | En r | x | 0.36 | 0.25 | 0.51 | 0.48 |
|  |  | y | 0.14 | 0.04 | 0.45 | 0.40 |  | y | 0.32 | 0.26 | 0.28 | 0.21 |  |  | y | 0.51 | 0.36 | 0.71 | 0.51 |  | y | 0.37 | 0.28 | 0.68 | 0.42 |
|  |  | z | 0.25 | 0.21 | 0.64 | 0.67 |  | z | 0.19 | 0.13 | 0.56 | 0.55 |  |  | z | 0.38 | 0.28 | 0.09 | 0.05 |  | z | 0.12 | 0.11 | 0.37 | 0.30 |
|  | Nt l | x | 0.26 | 0.14 | 0.51 | 0.18 | Nt l | x | 0.31 | 0.25 | 0.50 | 0.35 |  | En l | x | 0.61 | 0.47 | 0.65 | 0.34 | En l | x | 0.51 | 0.36 | 0.88 | 0.76 |
|  |  | y | 0.23 | 0.14 | 0.40 | 0.26 |  | y | 0.28 | 0.21 | 0.68 | 0.47 |  |  | y | 0.47 | 0.31 | 0.77 | 0.73 |  | y | 0.41 | 0.35 | 0.59 | 0.41 |
|  |  | z | 0.19 | 0.17 | 0.68 | 0.34 |  | z | 0.63 | 0.53 | 0.10 | 0.06 |  |  | z | 0.35 | 0.18 | 0.50 | 0.36 |  | z | 0.11 | 0.14 | 0.47 | 0.32 |
|  | Nm l | x | 0.27 | 0.19 | 0.53 | 0.18 | Cc | x | 0.58 | 0.54 | 0.71 | 0.62 |  | Em r | x | 0.52 | 0.43 | 0.90 | 0.39 | Em r | x | 0.65 | 0.25 | 0.76 | 0.36 |
|  |  | y | 0.17 | 0.14 | 0.37 | 0.18 |  | y | 0.43 | 0.33 | 0.55 | 0.34 |  |  | y | 0.69 | 0.14 | 0.53 | 0.34 |  | y | 0.74 | 0.66 | 0.93 | 0.57 |
|  |  | z | 0.30 | 0.24 | 0.71 | 0.50 |  | z | 0.21 | 0.19 | 0.24 | 0.17 |  |  | z | 0.35 | 0.25 | 0.45 | 0.10 |  | z | 0.40 | 0.36 | 0.59 | 0.44 |
|  | Nm r | x | 0.30 | 0.21 | 0.35 | 0.27 | Cm | x | 0.28 | 0.12 | 0.38 | 0.27 |  | Em l | x | 0.87 | 0.87 | 1.24 | 1.00 | Em l | x | 0.82 | 0.76 | 1.02 | 0.64 |
|  |  | y | 0.07 | 0.07 | 0.49 | 0.38 |  | y | 0.43 | 0.19 | 0.74 | 0.60 |  |  | y | 0.44 | 0.32 | 0.98 | 0.63 |  | y | 0.60 | 0.55 | 0.84 | 0.53 |
|  |  | z | 0.34 | 0.25 | 0.75 | 0.71 |  | z | 0.54 | 0.23 | 0.61 | 0.49 |  |  | z | 0.42 | 0.25 | 0.46 | 0.40 |  | z | 0.34 | 0.29 | 0.65 | 0.47 |
|  | G | x | 0.27 | 0.18 | 0.56 | 0.42 | G | x | 0.43 | 0.27 | 0.91 | 0.68 | Mouth | Sto | x | 0.59 | 0.33 | 0.59 | 0.56 | Sto | x | 0.44 | 0.30 | 0.62 | 0.52 |
|  |  | y | 0.20 | 0.13 | 0.83 | 0.44 |  | y | 0.51 | 0.50 | 0.44 | 0.42 |  |  | y | 0.43 | 0.28 | 0.59 | 0.37 |  | y | 0.61 | 0.31 | 0.82 | 0.64 |
|  |  | z | 0.42 | 0.28 | 0.34 | 0.26 |  | z | 0.36 | 0.33 | 0.42 | 0.25 |  |  | z | 0.18 | 0.16 | 0.33 | 0.32 |  | z | 0.32 | 0.20 | 0.42 | 0.55 |
|  | Prn | x | 0.35 | 0.23 | 0.79 | 0.51 | Prn | x | 0.54 | 0.34 | 0.50 | 0.48 |  | Li | x | 0.59 | 0.50 | 0.67 | 0.37 | Li | x | 0.50 | 0.47 | 0.71 | 0.38 |
|  |  | y | 0.27 | 0.12 | 0.88 | 0.77 |  | y | 0.47 | 0.37 | 0.20 | 0.21 |  |  | y | 0.45 | 0.38 | 0.65 | 0.29 |  | y | 0.64 | 0.68 | 0.88 | 0.59 |
|  |  | z | 0.30 | 0.26 | 0.33 | 0.23 |  | z | 0.28 | 0.24 | 0.96 | 0.74 |  |  | z | 0.09 | 0.06 | 0.36 | 0.29 |  | z | 0.23 | 0.29 | 0.28 | 0.24 |
|  | Cm | x | 0.30 | 0.27 | 0.68 | 0.58 | Al l | x | 0.47 | 0.27 | 0.89 | 0.75 |  | Ls | x | 0.53 | 0.36 | 0.61 | 0.45 | Ls | x | 0.62 | 0.44 | 0.62 | 0.50 |
|  |  | y | 0.41 | 0.24 | 0.99 | 0.68 |  | y | 0.62 | 0.94 | 0.26 | 0.20 |  |  | y | 0.52 | 0.42 | 0.57 | 0.42 |  | y | 0.72 | 0.49 | 0.87 | 0.70 |
|  |  | z | 0.34 | 0.22 | 0.32 | 0.23 |  | z | 0.09 | 0.07 | 0.64 | 0.49 |  |  | z | 0.15 | 0.15 | 0.60 | 0.10 |  | z | 0.48 | 0.49 | 0.50 | 0.23 |
|  | Stb | x | 0.41 | 0.31 | 0.92 | 0.63 | Al r | x | 0.59 | 0.53 | 0.81 | 0.49 |  | Sm | x | 0.67 | 0.57 | 0.82 | 0.45 | Sm | x | 0.78 | 0.57 | 0.97 | 0.68 |
|  |  | y | 0.52 | 0.39 | 0.81 | 0.66 |  | y | 0.59 | 0.39 | 0.67 | 0.46 |  |  | y | 0.79 | 0.52 | 0.71 | 0.89 |  | y | 0.79 | 0.57 | 1.00 | 0.57 |
|  |  | z | 0.08 | 0.08 | 0.59 | 0.33 |  | z | 0.54 | 0.46 | 0.63 | 0.36 |  |  | z | 0.14 | 0.12 | 0.37 | 0.35 |  | z | 0.10 | 0.10 | 0.23 | 0.12 |
|  | TDP r | x | 0.60 | 0.58 | 0.48 | 0.31 | TDP r | x | 0.50 | 0.44 | 0.61 | 0.38 |  | Sl | x | 0.91 | 0.21 | 1.02 | 0.32 | Sl | x | 0.91 | 0.60 | 0.97 | 0.61 |
|  |  | y | 0.32 | 0.25 | 1.37 | 1.08 |  | y | 0.57 | 0.43 | 1.05 | 0.90 |  |  | y | 0.90 | 0.75 | 0.95 | 0.69 |  | y | 0.74 | 0.71 | 0.82 | 0.52 |
|  |  | z | 0.33 | 0.29 | 0.09 | 0.07 |  | z | 0.69 | 0.63 | 0.20 | 0.17 |  |  | z | 0.18 | 0.18 | 0.48 | 0.41 |  | z | 0.91 | 0.60 | 1.05 | 0.23 |
| Nose | TDP l | x | 0.53 | 0.50 | 0.40 | 0.28 | TDP l | x | 0.66 | 0.51 | 0.75 | 0.72 |  | Pg | x | 0.80 | 0.57 | 1.18 | 0.76 | Pg | x | 1.01 | 0.77 | 1.02 | 0.89 |
|  |  | y | 0.45 | 0.35 | 1.16 | 0.79 |  | y | 0.68 | 0.60 | 0.73 | 0.65 |  |  | y | 1.27 | 0.97 | 2.00 | 0.53 |  | y | 1.31 | 1.02 | 1.65 | 0.54 |
|  |  | z | 0.31 | 0.29 | 0.81 | 0.66 |  | z | 0.60 | 0.55 | 0.74 | 0.71 |  |  | z | 0.78 | 0.54 | 0.94 | 0.64 |  | z | 1.28 | 1.25 | 1.16 | 0.86 |
|  | Se | x | 0.42 | 0.33 | 1.23 | 1.13 | Se | x | 0.59 | 0.51 | 0.65 | 0.46 |  | Me | x | 1.06 | 0.99 | 1.64 | 0.92 | Me | x | 2.06 | 1.56 | 2.11 | 1.34 |
|  |  | y | 0.70 | 0.58 | 0.41 | 0.34 |  | y | 0.73 | 0.57 | 0.78 | 0.60 |  |  | y | 0.87 | 0.79 | 0.81 | 0.72 |  | y | 0.71 | 0.58 | 0.97 | 0.71 |
|  |  | z | 0.17 | 0.15 | 0.79 | 0.55 |  | z | 0.70 | 0.79 | 0.84 | 0.66 |  |  | z | 1.34 | 0.94 | 1.95 | 1.81 |  | z | 1.41 | 1.04 | 1.61 | 0.95 |
|  | Cc | x | 0.63 | 0.41 | 1.08 | 0.64 | Nm r | x | 0.60 | 0.52 | 0.90 | 0.68 |  | C | x | 1.22 | 0.55 | 1.13 | 0.74 | C | x | 1.15 | 0.95 | 1.51 | 0.95 |
|  |  | y | 0.67 | 0.53 | 1.06 | 0.61 |  | y | 0.96 | 0.64 | 0.81 | 0.75 |  |  | y | 1.05 | 0.63 | 1.81 | 1.06 |  | y | 1.06 | 0.77 | 1.69 | 0.54 |
|  |  | z | 0.10 | 0.08 | 0.47 | 0.38 |  | z | 0.38 | 0.24 | 0.55 | 0.42 |  |  | z | 1.98 | 1.20 | 2.16 | 1.67 |  | z | 2.11 | 1.07 | 1.95 | 0.47 |
|  | Ac l | x | 0.53 | 0.37 | 0.80 | 0.76 | Nm l | x | 0.55 | 0.42 | 0.66 | 0.57 | Ear | Trg l | x | 0.36 | 0.35 | 0.91 | 0.75 | Trg l | x | 0.30 | 0.24 | 0.40 | 0.35 |
|  |  | y | 0.62 | 0.50 | 1.03 | 0.75 |  | y | 0.98 | 0.90 | 0.73 | 0.64 |  |  | y | 0.79 | 0.60 | 1.34 | 1.55 |  | y | 1.08 | 0.87 | 0.97 | 0.73 |
|  |  | z | 0.76 | 0.53 | 0.93 | 0.65 |  | z | 0.50 | 0.30 | 0.92 | 0.49 |  |  | z | 1.06 | 0.74 | 1.26 | 1.13 |  | z | 1.08 | 0.80 | 1.49 | 0.99 |
|  | Holn | x | 0.57 | 0.35 | 1.07 | 0.89 | Holn | x | 0.57 | 0.58 | 0.46 | 0.29 |  | Trg r | x | 0.40 | 0.36 | 0.67 | 0.62 | Trg r | x | 0.27 | 0.54 | 0.64 | 0.52 |
|  |  | y | 0.85 | 0.66 | 0.82 | 0.58 |  | y | 1.02 | 0.92 | 1.45 | 1.06 |  |  | y | 0.84 | 0.68 | 1.43 | 0.73 |  | y | 0.86 | 0.75 | 1.28 | 0.90 |
|  |  | z | 0.57 | 0.31 | 1.09 | 0.55 |  | z | 0.66 | 0.55 | 0.76 | 0.63 |  |  | z | 1.19 | 0.88 | 1.58 | 1.50 |  | z | 1.24 | 0.92 | 1.55 | 1.12 |
|  | Ac r | x | 0.57 | 0.39 | 1.05 | 0.77 | Ac r | x | 0.65 | 0.39 | 0.82 | 0.69 |  | Pa l | x | 0.65 | 0.62 | 1.41 | 0.44 | Pa l | x | 0.32 | 0.21 | 0.55 | 0.51 |
|  |  | y | 0.78 | 0.59 | 0.99 | 0.78 |  | y | 1.12 | 0.90 | 1.14 | 0.83 |  |  | y | 1.26 | 0.77 | 1.85 | 0.83 |  | y | 1.22 | 0.69 | 1.80 | 1.21 |
|  |  | z | 0.68 | 0.48 | 0.98 | 0.71 |  | z | 0.74 | 0.62 | 0.97 | 0.59 |  |  | z | 0.64 | 0.45 | 1.48 | 0.61 |  | z | 0.88 | 0.63 | 1.70 | 0.93 |
|  | Se'r | x | 0.60 | 0.53 | 0.97 | 0.74 | Se'r | x | 0.76 | 0.59 | 0.69 | 0.57 |  | Pa r | x | 0.63 | 0.47 | 0.97 | 0.36 | Pa r | x | 0.27 | 0.28 | 0.62 | 0.49 |
|  |  | y | 0.73 | 0.55 | 0.99 | 0.79 |  | y | 1.39 | 0.64 | 1.58 | 1.29 |  |  | y | 1.57 | 0.95 | 1.75 | 0.91 |  | y | 1.48 | 1.43 | 1.84 | 1.12 |
|  |  | z | 0.72 | 0.55 | 1.12 | 0.61 |  | z | 0.25 | 0.16 | 0.13 | 0.08 |  |  | z | 0.76 | 0.57 | 1.99 | 0.66 |  | z | 0.76 | 0.50 | 1.44 | 0.97 |
|  | Se'l | x | 0.34 | 0.24 | 1.05 | 0.69 | Se'l | x | 0.69 | 0.47 | 1.16 | 1.12 | Other | Tri | x | 1.03 | 1.05 | 1.60 | 1.15 | Tri | x | 1.01 | 0.78 | 1.11 | 0.93 |
|  |  | y | 1.06 | 1.09 | 1.11 | 0.74 |  | y | 1.35 | 1.11 | 0.85 | 0.69 |  |  | y | 1.99 | 1.13 | 2.13 | 1.10 |  | y | 1.43 | 1.32 | 1.86 | 1.15 |
|  |  | z | 0.58 | 0.40 | 1.03 | 0.78 |  | z | 0.60 | 0.49 | 1.15 | 0.95 |  |  | z | 1.37 | 1.12 | 2.04 | 0.87 |  | z | 1.22 | 0.61 | 1.21 | 0.87 |
|  | N | x | 0.72 | 0.40 | 0.99 | 0.57 | N | x | 1.01 | 0.65 | 0.70 | 0.52 |  | Zy r | x | 1.31 | 0.90 | 1.61 | 1.03 | Zy r | x | 2.02 | 0.61 | 1.63 | 0.99 |
|  |  | y | 1.03 | 0.66 | 1.36 | 0.53 |  | y | 1.05 | 0.62 | 1.46 | 0.99 |  |  | y | 2.08 | 1.17 | 2.21 | 1.11 |  | y | 2.13 | 1.52 | 2.57 | 1.28 |
|  |  | z | 0.08 | 0.06 | 0.77 | 0.39 |  | z | 0.76 | 0.52 | 0.93 | 1.00 |  |  | z | 0.98 | 0.61 | 2.11 | 1.03 |  | z | 1.86 | 0.89 | 2.05 | 1.56 |
|  | Al l | x | 0.60 | 0.60 | 1.14 | 0.72 | Ac l | x | 0.80 | 0.57 | 0.99 | 0.43 |  | Zy l | x | 1.32 | 1.07 | 3.09 | 1.46 | Zy l | x | 1.65 | 1.02 | 1.55 | 0.73 |
|  |  | y | 0.29 | 0.24 | 0.56 | 0.34 |  | y | 1.34 | 0.42 | 1.44 | 0.76 |  |  | y | 1.98 | 1.40 | 2.14 | 0.95 |  | y | 2.47 | 1.58 | 2.83 | 1.16 |
|  |  | z | 1.12 | 0.83 | 1.39 | 0.87 |  | z | 0.50 | 0.38 | 0.71 | 0.18 |  |  | z | 2.00 | 1.61 | 2.45 | 1.10 |  | z | 1.91 | 1.09 | 2.05 | 1.37 |

**Supplementary Table 2. Overall evaluation of the reproducibility of female and male facial soft tissue landmarks on three spatial planes**

| Gender | Female | | | | | | Male | | | | | | Gender | Female | | | | | | Male | | | | | |
| --- | --- | --- | --- | --- | --- | --- | --- | --- | --- | --- | --- | --- | --- | --- | --- | --- | --- | --- | --- | --- | --- | --- | --- | --- | --- |
| Area | Landmarks | Axis | Intra-rater | | Inter-rater | | Landmarks | Axis | Intra-rater | | Inter-rater | | Area | Landmarks | Axis | Intra-rater | | Inter-rater | | Landmarks | Axis | Intra-rater | | Inter-rater | |
|  |  |  | Mean | SD | Mean | SD |  |  | Mean | SD | Mean | SD |  |  |  | Mean | SD | Mean | SD |  |  | Mean | SD | Mean | SD |
| Nose | Prn | x | 0.22 | 0.19 | 0.35 | 0.28 | Prn | x | 0.23 | 0.19 | 0.30 | 0.23 | Nose | Al r | x | 0.82 | 0.43 | 1.08 | 0.86 | Al r | x | 0.60 | 0.48 | 1.26 | 1.11 |
|  |  | y | 0.30 | 0.26 | 0.34 | 0.18 |  | y | 0.23 | 0.23 | 0.30 | 0.17 |  |  | y | 0.96 | 0.67 | 2.29 | 1.41 |  | y | 1.01 | 0.77 | 2.00 | 1.42 |
|  |  | z | 0.20 | 0.22 | 0.27 | 0.13 |  | z | 0.16 | 0.13 | 0.33 | 0.16 |  |  | z | 0.74 | 0.55 | 0.97 | 0.63 |  | z | 0.90 | 0.57 | 1.08 | 0.69 |
|  | Cm | x | 0.28 | 0.25 | 0.26 | 0.19 | Cm | x | 0.26 | 0.22 | 0.28 | 0.11 |  | Al l | x | 0.76 | 0.52 | 0.96 | 0.73 | Al l | x | 0.80 | 0.68 | 1.63 | 1.14 |
|  |  | y | 0.23 | 0.21 | 0.28 | 0.24 |  | y | 0.24 | 0.19 | 0.30 | 0.13 |  |  | y | 1.09 | 1.16 | 2.48 | 1.53 |  | y | 1.01 | 0.41 | 0.93 | 0.85 |
|  |  | z | 0.22 | 0.17 | 0.46 | 0.35 |  | z | 0.17 | 0.17 | 0.37 | 0.17 |  |  | z | 0.67 | 0.56 | 0.82 | 0.59 |  | z | 0.74 | 0.50 | 1.83 | 1.01 |
|  | Holn | x | 0.29 | 0.16 | 0.44 | 0.30 | Holn | x | 0.24 | 0.16 | 0.25 | 0.08 |  | N | x | 0.91 | 0.66 | 1.37 | 0.62 | N | x | 1.02 | 0.93 | 1.01 | 1.16 |
|  |  | y | 0.24 | 0.14 | 0.29 | 0.22 |  | y | 0.20 | 0.13 | 0.29 | 0.13 |  |  | y | 1.02 | 0.63 | 1.90 | 0.88 |  | y | 1.12 | 1.02 | 1.87 | 0.87 |
|  |  | z | 0.21 | 0.18 | 0.32 | 0.27 |  | z | 0.24 | 0.21 | 0.43 | 0.18 |  |  | z | 0.76 | 0.52 | 1.21 | 0.54 |  | z | 0.79 | 0.47 | 0.98 | 0.54 |
|  | TDP r | x | 0.35 | 0.21 | 0.32 | 0.28 | TDP r | x | 0.26 | 0.21 | 0.34 | 0.19 |  | Stb | x | 1.66 | 1.25 | 1.82 | 0.68 | Stb | x | 1.86 | 1.39 | 1.13 | 0.58 |
|  |  | y | 0.10 | 0.10 | 0.24 | 0.21 |  | y | 0.29 | 0.09 | 0.39 | 0.23 |  |  | y | 1.70 | 1.08 | 2.33 | 1.28 |  | y | 0.96 | 0.67 | 2.13 | 1.09 |
|  |  | z | 0.38 | 0.31 | 0.45 | 0.25 |  | z | 0.27 | 0.24 | 0.33 | 0.17 |  |  | z | 1.26 | 0.58 | 1.36 | 0.64 |  | z | 1.38 | 0.38 | 1.55 | 0.47 |
|  | TDP l | x | 0.29 | 0.20 | 0.42 | 0.25 | TDP l | x | 0.32 | 0.39 | 0.31 | 0.31 | Eye | Em l | x | 0.34 | 0.24 | 0.43 | 0.34 | Em l | x | 0.44 | 0.27 | 0.48 | 0.39 |
|  |  | y | 0.09 | 0.07 | 0.40 | 0.39 |  | y | 0.30 | 0.69 | 0.73 | 0.53 |  |  | y | 0.36 | 0.32 | 0.62 | 0.48 |  | y | 0.39 | 0.30 | 0.67 | 0.45 |
|  |  | z | 0.51 | 1.56 | 0.77 | 0.70 |  | z | 0.27 | 0.44 | 0.42 | 0.41 |  |  | z | 0.19 | 0.20 | 0.19 | 0.16 |  | z | 0.30 | 0.29 | 0.22 | 0.21 |
|  | Se | x | 0.39 | 0.32 | 0.47 | 0.31 | Se | x | 0.40 | 0.44 | 0.33 | 0.24 |  | Em r | x | 0.45 | 0.37 | 0.71 | 0.38 | Em r | x | 0.58 | 0.41 | 0.67 | 0.76 |
|  |  | y | 0.44 | 0.43 | 0.91 | 0.07 |  | y | 0.14 | 0.13 | 0.67 | 0.46 |  |  | y | 0.43 | 0.39 | 0.55 | 0.42 |  | y | 0.41 | 0.29 | 0.62 | 0.42 |
|  |  | z | 0.32 | 0.38 | 0.10 | 0.09 |  | z | 0.32 | 0.30 | 0.51 | 0.35 |  |  | z | 0.27 | 0.32 | 0.25 | 0.25 |  | z | 0.24 | 0.23 | 0.32 | 0.29 |
|  | G | x | 0.48 | 0.30 | 0.78 | 0.63 | G | x | 0.33 | 0.33 | 0.50 | 0.35 |  | En r | x | 0.58 | 0.53 | 0.71 | 0.67 | En r | x | 0.88 | 0.60 | 0.96 | 1.12 |
|  |  | y | 0.47 | 0.40 | 0.25 | 0.24 |  | y | 0.37 | 0.21 | 0.84 | 0.68 |  |  | y | 0.48 | 0.36 | 0.74 | 0.66 |  | y | 0.64 | 0.54 | 0.74 | 0.70 |
|  |  | z | 0.09 | 0.13 | 0.90 | 0.68 |  | z | 0.25 | 0.13 | 0.09 | 0.06 |  |  | z | 0.35 | 0.25 | 0.67 | 0.42 |  | z | 0.40 | 0.30 | 0.64 | 0.53 |
|  | Ac l | x | 0.22 | 0.22 | 0.92 | 0.74 | Ac l | x | 0.28 | 0.22 | 0.72 | 0.79 |  | En l | x | 0.62 | 0.48 | 0.69 | 0.81 | En l | x | 0.96 | 0.80 | 0.97 | 0.25 |
|  |  | y | 0.15 | 0.09 | 0.27 | 0.18 |  | y | 0.19 | 0.66 | 0.65 | 0.38 |  |  | y | 0.58 | 0.53 | 0.86 | 0.71 |  | y | 0.52 | 0.40 | 0.85 | 0.60 |
|  |  | z | 0.61 | 1.58 | 0.93 | 0.48 |  | z | 0.52 | 0.35 | 0.35 | 0.31 |  |  | z | 0.35 | 0.27 | 0.73 | 0.42 |  | z | 0.45 | 0.33 | 0.72 | 0.51 |
|  | Ac r | x | 0.41 | 0.25 | 0.84 | 0.66 | Ac r | x | 0.40 | 0.27 | 0.75 | 0.70 | Mouth | Ls | x | 0.60 | 0.53 | 0.68 | 0.63 | Ls | x | 0.51 | 0.35 | 0.50 | 0.43 |
|  |  | y | 0.24 | 0.17 | 0.88 | 0.46 |  | y | 0.52 | 0.48 | 0.53 | 0.54 |  |  | y | 0.40 | 0.27 | 0.54 | 0.32 |  | y | 0.46 | 0.33 | 0.67 | 1.12 |
|  |  | z | 0.56 | 0.26 | 0.55 | 0.42 |  | z | 0.07 | 0.06 | 0.85 | 0.63 |  |  | z | 0.20 | 0.19 | 0.27 | 0.18 |  | z | 0.19 | 0.16 | 0.25 | 0.17 |
|  | Nt l | x | 0.31 | 0.21 | 0.46 | 0.29 | Nt l | x | 0.34 | 0.13 | 0.79 | 0.41 |  | Li | x | 0.58 | 0.50 | 0.62 | 0.25 | Li | x | 0.44 | 0.31 | 0.61 | 0.54 |
|  |  | y | 0.28 | 0.17 | 0.92 | 0.64 |  | y | 0.40 | 0.19 | 0.87 | 0.67 |  |  | y | 0.63 | 0.62 | 0.80 | 0.49 |  | y | 0.39 | 0.37 | 0.45 | 0.51 |
|  |  | z | 0.62 | 0.51 | 0.91 | 0.70 |  | z | 0.43 | 0.31 | 0.41 | 0.21 |  |  | z | 0.26 | 0.27 | 0.32 | 0.11 |  | z | 0.47 | 0.09 | 0.50 | 0.19 |
|  | Nt r | x | 0.40 | 0.30 | 0.73 | 0.52 | Nt r | x | 0.37 | 0.22 | 1.06 | 0.68 |  | Sto | x | 0.57 | 0.41 | 0.62 | 0.43 | Sto | x | 0.57 | 0.52 | 0.67 | 0.32 |
|  |  | y | 0.53 | 0.49 | 0.96 | 0.63 |  | y | 0.40 | 0.26 | 0.56 | 0.39 |  |  | y | 0.71 | 0.46 | 0.92 | 0.69 |  | y | 0.46 | 0.42 | 0.60 | 0.29 |
|  |  | z | 0.43 | 0.38 | 0.79 | 0.50 |  | z | 0.43 | 0.20 | 0.50 | 0.27 |  |  | z | 0.45 | 0.49 | 0.39 | 0.30 |  | z | 0.24 | 0.17 | 0.27 | 0.15 |
|  | Nl l | x | 0.35 | 0.31 | 0.92 | 0.65 | Nl l | x | 0.45 | 0.50 | 0.96 | 0.82 |  | Me | x | 0.50 | 0.37 | 0.50 | 0.39 | Me | x | 0.43 | 0.30 | 0.50 | 0.32 |
|  |  | y | 0.47 | 0.40 | 0.56 | 0.39 |  | y | 0.55 | 0.55 | 0.61 | 0.38 |  |  | y | 0.79 | 0.74 | 1.12 | 0.77 |  | y | 0.61 | 0.64 | 0.95 | 0.64 |
|  |  | z | 0.57 | 0.55 | 0.98 | 0.68 |  | z | 0.37 | 0.25 | 1.04 | 0.65 |  |  | z | 0.46 | 0.48 | 0.73 | 0.53 |  | z | 0.43 | 0.32 | 0.48 | 0.43 |
|  | Nl r | x | 0.54 | 0.40 | 0.91 | 0.65 | Nl r | x | 0.59 | 0.49 | 0.76 | 0.60 |  | Sl | x | 0.46 | 0.28 | 0.96 | 0.56 | Sl | x | 0.86 | 0.78 | 1.25 | 0.87 |
|  |  | y | 0.53 | 0.54 | 0.93 | 0.85 |  | y | 0.44 | 0.53 | 0.74 | 0.67 |  |  | y | 0.88 | 0.55 | 1.50 | 1.26 |  | y | 0.68 | 0.51 | 0.75 | 0.34 |
|  |  | z | 0.54 | 0.45 | 0.95 | 0.72 |  | z | 0.44 | 0.56 | 1.25 | 1.14 |  |  | z | 0.76 | 0.84 | 0.30 | 0.28 |  | z | 0.49 | 0.08 | 0.90 | 0.36 |
|  | Ort l | x | 0.53 | 0.54 | 1.42 | 0.92 | Ort l | x | 0.54 | 0.30 | 1.35 | 1.32 |  | Pg | x | 0.78 | 0.58 | 0.57 | 0.46 | Pg | x | 0.80 | 0.61 | 0.91 | 0.84 |
|  |  | y | 0.56 | 0.40 | 0.62 | 0.43 |  | y | 0.48 | 0.35 | 0.77 | 0.56 |  |  | y | 0.71 | 0.29 | 1.36 | 1.75 |  | y | 0.89 | 0.56 | 1.47 | 0.86 |
|  |  | z | 0.62 | 0.38 | 0.50 | 0.34 |  | z | 0.52 | 0.22 | 0.86 | 0.79 |  |  | z | 0.68 | 0.29 | 1.18 | 0.99 |  | z | 0.18 | 0.15 | 0.23 | 0.18 |
|  | Ort r | x | 0.64 | 0.31 | 0.59 | 0.42 | Ort r | x | 0.53 | 0.42 | 0.82 | 0.59 |  | Sm | x | 0.83 | 0.52 | 1.55 | 0.86 | Sm | x | 0.88 | 0.65 | 1.70 | 0.74 |
|  |  | y | 0.53 | 0.33 | 1.51 | 0.94 |  | y | 0.45 | 0.37 | 0.80 | 0.58 |  |  | y | 1.28 | 1.09 | 0.95 | 0.81 |  | y | 1.30 | 0.88 | 0.83 | 0.60 |
|  |  | z | 0.59 | 0.26 | 0.27 | 0.23 |  | z | 0.60 | 0.27 | 1.39 | 0.97 |  |  | z | 1.23 | 1.26 | 1.89 | 1.36 |  | z | 0.83 | 0.55 | 1.41 | 0.83 |
| Nose | Cc | x | 0.47 | 0.27 | 0.88 | 0.72 | Cc | x | 0.63 | 0.56 | 1.01 | 0.89 |  | C | x | 1.13 | 0.77 | 1.14 | 0.67 | C | x | 0.98 | 0.96 | 1.39 | 1.00 |
|  |  | y | 0.94 | 0.65 | 1.14 | 0.88 |  | y | 0.84 | 0.73 | 1.26 | 0.88 |  |  | y | 0.85 | 0.65 | 0.92 | 0.72 |  | y | 0.98 | 0.77 | 1.35 | 1.02 |
|  |  | z | 0.42 | 0.14 | 0.78 | 0.64 |  | z | 0.20 | 0.19 | 1.07 | 1.27 |  |  | z | 1.81 | 1.04 | 2.81 | 1.77 |  | z | 1.26 | 0.72 | 1.90 | 0.95 |
|  | Sn | x | 0.82 | 0.62 | 0.68 | 0.49 | Sn | x | 0.50 | 0.33 | 0.64 | 0.49 | Ear | Pa l | x | 0.29 | 0.22 | 0.83 | 0.61 | Pa l | x | 0.34 | 0.15 | 0.55 | 0.28 |
|  |  | y | 0.75 | 0.67 | 0.87 | 0.63 |  | y | 0.91 | 0.87 | 1.17 | 0.40 |  |  | y | 0.85 | 0.64 | 1.59 | 1.29 |  | y | 1.01 | 0.91 | 1.42 | 0.88 |
|  |  | z | 0.25 | 0.41 | 0.97 | 0.14 |  | z | 0.38 | 0.34 | 1.06 | 0.74 |  |  | z | 1.04 | 0.60 | 1.63 | 1.20 |  | z | 0.84 | 0.60 | 1.19 | 0.69 |
|  | Se'l | x | 0.51 | 0.42 | 0.74 | 0.57 | Se‘l | x | 0.43 | 0.42 | 0.49 | 0.28 |  | Pa r | x | 0.18 | 0.14 | 0.73 | 0.49 | Pa r | x | 0.30 | 0.30 | 0.52 | 0.33 |
|  |  | y | 0.97 | 0.82 | 0.84 | 0.68 |  | y | 0.98 | 1.05 | 1.91 | 1.53 |  |  | y | 0.84 | 0.75 | 2.31 | 1.10 |  | y | 0.91 | 0.73 | 1.24 | 0.68 |
|  |  | z | 0.30 | 0.24 | 1.42 | 0.67 |  | z | 0.35 | 0.19 | 0.38 | 0.26 |  |  | z | 1.24 | 0.97 | 1.51 | 1.11 |  | z | 1.09 | 0.80 | 1.43 | 0.86 |
|  | Se'r | x | 0.56 | 0.37 | 0.59 | 0.37 | Nb l | x | 0.49 | 0.38 | 0.55 | 0.53 |  | Trg r | x | 0.49 | 0.59 | 0.82 | 0.63 | Trg r | x | 0.36 | 0.46 | 0.38 | 0.26 |
|  |  | y | 0.69 | 0.22 | 1.75 | 1.02 |  | y | 0.64 | 0.45 | 1.98 | 0.61 |  |  | y | 1.29 | 0.60 | 1.45 | 0.52 |  | y | 1.24 | 0.95 | 1.53 | 0.86 |
|  |  | z | 0.85 | 0.90 | 0.35 | 0.29 |  | z | 0.96 | 0.60 | 0.27 | 0.24 |  |  | z | 1.16 | 0.69 | 1.81 | 0.69 |  | z | 0.78 | 0.45 | 1.22 | 0.75 |
|  | Nb l | x | 0.78 | 0.71 | 0.42 | 0.35 | Nb r | x | 0.59 | 0.50 | 0.69 | 0.53 |  | Trg l | x | 0.65 | 0.50 | 0.69 | 0.55 | Trg l | x | 0.74 | 0.77 | 0.33 | 0.30 |
|  |  | y | 0.70 | 1.02 | 0.98 | 0.63 |  | y | 0.64 | 0.56 | 1.55 | 0.99 |  |  | y | 1.78 | 0.78 | 2.62 | 1.28 |  | y | 1.60 | 1.26 | 1.75 | 1.20 |
|  |  | z | 0.71 | 0.61 | 1.64 | 0.81 |  | z | 0.91 | 0.75 | 1.36 | 0.98 |  |  | z | 0.94 | 0.74 | 2.12 | 0.80 |  | z | 0.90 | 0.78 | 1.77 | 0.98 |
|  | Nb r | x | 0.53 | 0.38 | 0.84 | 0.96 | Se'r | x | 0.85 | 0.55 | 1.65 | 1.32 | Other | Tri | x | 1.15 | 0.63 | 1.20 | 0.56 | Tri | x | 1.44 | 0.76 | 1.51 | 0.82 |
|  |  | y | 0.70 | 0.51 | 1.19 | 0.83 |  | y | 0.96 | 0.62 | 1.03 | 0.77 |  |  | y | 1.78 | 1.12 | 1.94 | 1.12 |  | y | 1.75 | 1.03 | 1.81 | 0.96 |
|  |  | z | 0.95 | 0.93 | 1.52 | 1.21 |  | z | 0.19 | 0.28 | 1.27 | 0.78 |  |  | z | 1.60 | 1.33 | 1.70 | 1.06 |  | z | 1.28 | 0.99 | 1.40 | 0.89 |
|  | Nm l | x | 0.90 | 0.58 | 0.98 | 0.59 | Nm l | x | 0.57 | 0.41 | 1.00 | 0.81 |  | Zy r | x | 1.36 | 0.92 | 1.73 | 1.11 | Zy r | x | 1.22 | 0.96 | 1.53 | 1.00 |
|  |  | y | 1.04 | 0.78 | 1.50 | 0.44 |  | y | 1.09 | 0.81 | 1.87 | 1.44 |  |  | y | 2.22 | 1.71 | 2.40 | 1.53 |  | y | 1.75 | 1.34 | 2.02 | 0.84 |
|  |  | z | 0.10 | 0.10 | 1.38 | 0.38 |  | z | 0.61 | 0.39 | 0.96 | 0.53 |  |  | z | 1.92 | 1.32 | 2.08 | 1.13 |  | z | 1.88 | 0.80 | 2.15 | 1.11 |
|  | Nm r | x | 0.72 | 0.50 | 1.00 | 0.64 | Nm r | x | 0.78 | 0.60 | 1.35 | 0.66 |  | Zy l | x | 1.55 | 1.12 | 2.06 | 1.25 | Zy l | x | 1.27 | 1.19 | 1.59 | 0.95 |
|  |  | y | 0.94 | 0.73 | 1.81 | 1.07 |  | y | 0.81 | 0.83 | 1.83 | 0.87 |  |  | y | 1.96 | 1.10 | 2.33 | 1.54 |  | y | 1.78 | 1.29 | 2.63 | 0.78 |
|  |  | z | 0.85 | 0.29 | 0.91 | 0.71 |  | z | 0.83 | 0.76 | 0.96 | 0.70 |  |  | z | 2.33 | 2.06 | 2.35 | 1.27 |  | z | 2.16 | 1.03 | 2.46 | 0.72 |
